# Cell transplantation: relevance in understanding brain development and prospects in brain repair

**DOI:** 10.3389/fncel.2012.00056

**Published:** 2012-11-23

**Authors:** Mohamed Jaber, Afsaneh Gaillard

**Affiliations:** Experimental and Clinical Neurosciences Laboratory, INSERM U1084, University of PoitiersPoitiers, France

Neurodegenerative disorders such as Parkinson's diseases (PD) and Huntington's diseases (HD), neuronal injuries following trauma and neuronal cell death following strokes are major debilitating affections that are often accompanied by motor and cognitive dysfunctions with limited treatment options. Cell transplantation therapies have been considered for the last three decades as a serious avenue to explore with the ultimate aim to replace lost neurons with “new ones” initially originating from fetal neuroblasts and recently deriving from various sources of stem cells.

One of the many factors affecting the success of cell transplantation therapies is host immune response to the graft. This came as an evidence when attempts of cell therapy was undertaken with the use of human fetal neuroblasts or porcine fetal neuronal tissue for a time envisaged as a potential useful cell source for xenotransplantation in the human brain. However, and as Bonnamain et al. (2012) describe in this issue, this avenue led to disappointing results which in turn led to a logical hold of this line of research.

Pauly et al. (2012) focus on GABAergic striatal neurons with a detailed review on their development and clinical applications in HD. They report that in animal models of HD, the success of cellular transplantation with embryonic striatal transplants is influenced by many parameters including the host environment before and after the transplantation as well as experience and training. From a clinical perspective, despite contrasting findings, recent reports indicate that HD patients that underwent cell transplantation showed motor and cognitive improvements.

Given that the use of fetal tissue as a cell source for neural transplantation is not without causing logistical and ethical problems, a significant research activity has been oriented toward finding alternative sources of neural cells. Among these, pluripotent stem cells seem to be an obvious choice as these cells are theoretically able to differentiate into any cell type of any organ. As Benchoua and Onteniente (2012) put it in their review, pluripotent stem cells “hold the potential to revolutionize the field of neurodegenerative medicine by offering a robust and flexible source of allogenic or HLA compatible neuronal precursors.” In their review, the authors focus on regional and local patterning of these cells to induce their differentiation into specific neural progenitors. Then, they discuss safety issues regarding these cells, factors that maintain their commitment after transplantation and facilitate their integration within the host brain, mostly in animal models of HD and PD. They also describe the recent attempts in clinical studies where trials with pluripotent stem cells are just beginning.

The paper of Denham et al. (2012) reviews intra cerebral transplantation of neurons generated from human embryonic stem (hES) cells in neonatal rats and focus on axonal growth in the host brain and the corresponding electrophysiological properties. They show that neurons generated from hES cells are capable of extensive growth within the host brain and display properties consistent with functional integration at the electrophysiological level. While these findings are encouraging, they need to be replicated in the context of adult brain repair.

García-Parra et al. (2012) propose a study presenting a new polymeric support able to induce neuronal differentiation in both PC12 cell line and adult primary skin-derived precursor cells *in vitro*. They detail the use of a combined photolithographic technique to create a topography of micropatterned substrates composed of extracellular matrix providing the cells with appropriate cues for their differentiation. Although these cellular models are far from mimicking the characteristics of neuronal cells *in vivo*, they can set the basis for new avenues to explore the differentiation of stem cells *in vitro* after adjustment of the proper microenvironment in order to obtain the requested specific neuronal subtype.

In the *in vivo* area, de Chevigny et al. (2012) present a very elegant study aimed at characterizing the spatial and temporal expression of two major transcription factors, Pax6 and DIx2 that are implicated in the generation of olfactory bulb (OB) neurons. OB neurogenesis attracts the attention of several laboratories as their dopamine neurons, or their precursors, are presented as of potential interest in cell replacement or recruitment therapies in PD. The dynamic expression data presented for these two transcription factors indicate that while Pax6 is implicated in OB dopaminergic cell fate in a specific and permanent manner, DIx2 expression is more generalized and transient. Besides a better explanation of factors involved in the cell lineage of dopamine neurons, this type of studies is helpful in identifying molecular mechanisms involved in neuronal subtype specification in the postnatal brain.

Following transplantation, axons derived from transplanted neurons need to find their way and innervate target areas. Our own findings showed that embryonic mesencephalic dopamine neurons transplanted in the substantia nigra in an animal model of PD are able to extend axons toward the striatum (Gaillard et al., 2009; Gaillard and Jaber, 2011). These results suggest that specific guidance cues exist in the adult brain and that axons from transplanted embryonic cells are able to respond to theses cues, guiding them to their final targets. The review by Prestoz et al. (2012) summarizes the current knowledge on the identity of cellular and molecular signals thought to be involved in development of the dopamine pathway during embryogenesis in the rodent central nervous system. The paper also describes the modulation of these factors following lesion and transplantation and their potential implication in restoring damaged pathways.

The review by Saha et al. (2012) is focused on stimulation, migration of the pools of neural stem or precursor cells, particularly in the subventricular zone following cortical injuries, and details the cellular and molecular mechanisms involved in these processes. These range from molecular factors, glial reaction, vasculature as well as physical exercise. The description is extended to future avenues that need to be explored in order to better induce these reactions, as their efficiency in brain repair is very limited.

Although cell transplantation in the damaged brain is not likely to be routinely performed in the near future, the different paths that are evoked in this series of reviews should yield safer, more effective and physiologically relevant transplantation procedures.
